# Near complete resolution of COVID-19 pneumonia lesions in a patient of carcinoma lung treated with volumetric modulated arc therapy

**DOI:** 10.1259/bjrcr.20210047

**Published:** 2021-07-28

**Authors:** Anil Kumar Anand, Priyanka Singh, Amit Kumar, Anil Kumar Bansal, Heigrujam Malhotra Singh, Tarun Sharma

**Affiliations:** 1 Department of Radiation Oncology, Max Institute of Cancer Care, Max Super Speciality Hospital, Saket, New Delhi, India; 2 Department of Imaging, Max Super Speciality Hospital, Saket, New Delhi, India; 3 Division of Medical Physics, Department of Radiation Oncology, Max Institute of Cancer Care, Max Super Speciality Hospital, Saket, New Delhi, India; 4 Department of Pulmonology, Max Super Speciality Hospital, Saket, New Delhi, India

## Abstract

A 49-year-old male presented with non-small cell lung cancer in right upper lobe lung with solitary brain metastasis. He developed COVID-19 infection and received domiciliary treatment for 3 weeks. Three weeks after testing negative for RT-PCR test, he received stereotactic radiosurgery (SRS) to brain metastasis. He then presented in emergency with pain in the epigastrium and was detected with amoebic liver abscess. Subsequently, he developed recurrent hemoptysis for which he was planned for palliative radiation to right lung mass. Planning CT scan showed COVID-19 pneumonia lesions involving bilateral lungs in addition to right upper lobe tumour. Palliative radiation 8 Gy/1 fraction was delivered to lung tumour with VMAT technique. He showed near total resolution of COVID-19 lesions with low-dose scatter radiation and relief of haemoptysis.

## Introduction

COVID-19 pandemic is still ranging the world with 163 million cases and 3.36 million deaths reported as on 18 May 2021.^1^ Lung cancer is the second most common cancer worldwide with 2.20 million cases and 1.7 million deaths in the year 2020.^2^ There are several challenges in the management of patients with lung cancer with co-existing COVID-19 pneumonia with higher morbidity and mortality reported in these patients.^3^ In one study, COVID-19 pneumonia was seen in 88% of patients at 6 weeks, which although improved over a period of next 12–24 weeks but still 56% of patients had persisting COVID-19 lung lesions at 3 months.^4^

Lung cancer patients usually present in locally advanced stages.^3^ These patients require radiation therapy as a radical treatment (± chemotherapy) or as palliative measure to relieve symptoms like cough, hemoptysis and breathlessness. The safety of delivering radiation to the lung tumour in a patient with COVID-19 pneumonia is not known, and there may be a concern that radiation may have deleterious effects on rest of the lung.

It has also been postulated that low radiation in the range of 0.25 Gy to 1 Gy has beneficial effects on COVID-19 pneumonia by inducing anti-inflammatory response.^5^ We report a case of non-small cell lung cancer with COVID-19 pneumonia where palliative radiation was delivered to right upper lobe tumour for recurrent hemoptysis.

## Case presentation

A 49-year-old gentleman presented with headache and vertigo of 3 days duration on 2 September 2020. He was a chronic smoker with chronic obstructive pulmonary disease (COPD) with no other co-morbidity. His routine laboratory investigations were normal. Contrast-enhanced MRI of the brain revealed left cerebellar enhancing mass 2.4 cm in diameter with gross perilesional oedema consistent with metastatic lesion. He underwent whole body 18F-FDG Positron Emission Tomography (PET)-CT scan, which revealed heterogeneously enhancing, FDG avid soft tissue mass lesion (measuring 5.9 × 4.4 cm) in right upper lobe apical segment. There were also subpleural fibrotic changes with paraseptal emphysematous bullae consistent with COPD. FDG avid thick rim enhancing lesion was seen in left cerebellum with perilesional oedema. No other metastatic deposits were seen elsewhere. Fibreoptic bronchoscopy revealed no endobronchial mass. Bronchoalveolar lavage for acid-fast bacillus (stain/culture), Gram staining and fungal examination were normal. CT-guided Trucut biopsy from right upper lobe lung lesion reported on 8 October 2020 as pulmonary adenosquamous carcinoma. Tumor cells expressed CK, TTF–1, CKT and Napsin. A diagnosis of adenosquamous carcinoma of right upper lobe lung with solitary brain metastasis was made.

He was planned for stereotactic Radiosurgery (SRS) to brain lesion but on routine screening as a departmental protocol, real-time PCR (RT-PCR) for COVID–19 came positive on 14 October 2020 and the procedure was abandoned. He remained in home isolation for 3 weeks and returned on 2 November 2020 with negative RT-PCR report. He only had mild cough and his routine haematology and biochemistry investigations were normal. Other markers like c-reactive protein, lactate dehydrogenase, D-dimer and Interleukin-6 were not done since he had no symptoms ascribed to COVID-19 infection. He received SRS to left cerebellar lesion delivering a dose of 30 Gy/5 frs from 2 to 7 November 2020. He tolerated SRS well and his neurological symptoms improved.

He presented in emergency after 1 week with severe pain in right side-of chest and upper abdomen. Clinical examination revealed bilateral decrease in air entry in lungs and crepitations. Chest X-ray revealed soft tissue lesion in right upper lobe with few linear and nodular opacities in bilateral upper zone of lungs. Ultrasound whole abdomen revealed multiple large heteroechoic areas in right lobe of liver largest measuring 7 × 6 cm in segment V/VII and confirmed to be liver abscesses on Triple-phase MRI Scan.

Ultrasound-guided biopsy from liver lesion was done with insertion of pigtail catheter. Amoebic serology was positive. Abscess fluid culture showed growth of klabsiella pneumoniae. He received injection metronidazole, ceftriaxone and tab hydroxychloroquin.

**Figure 1.**
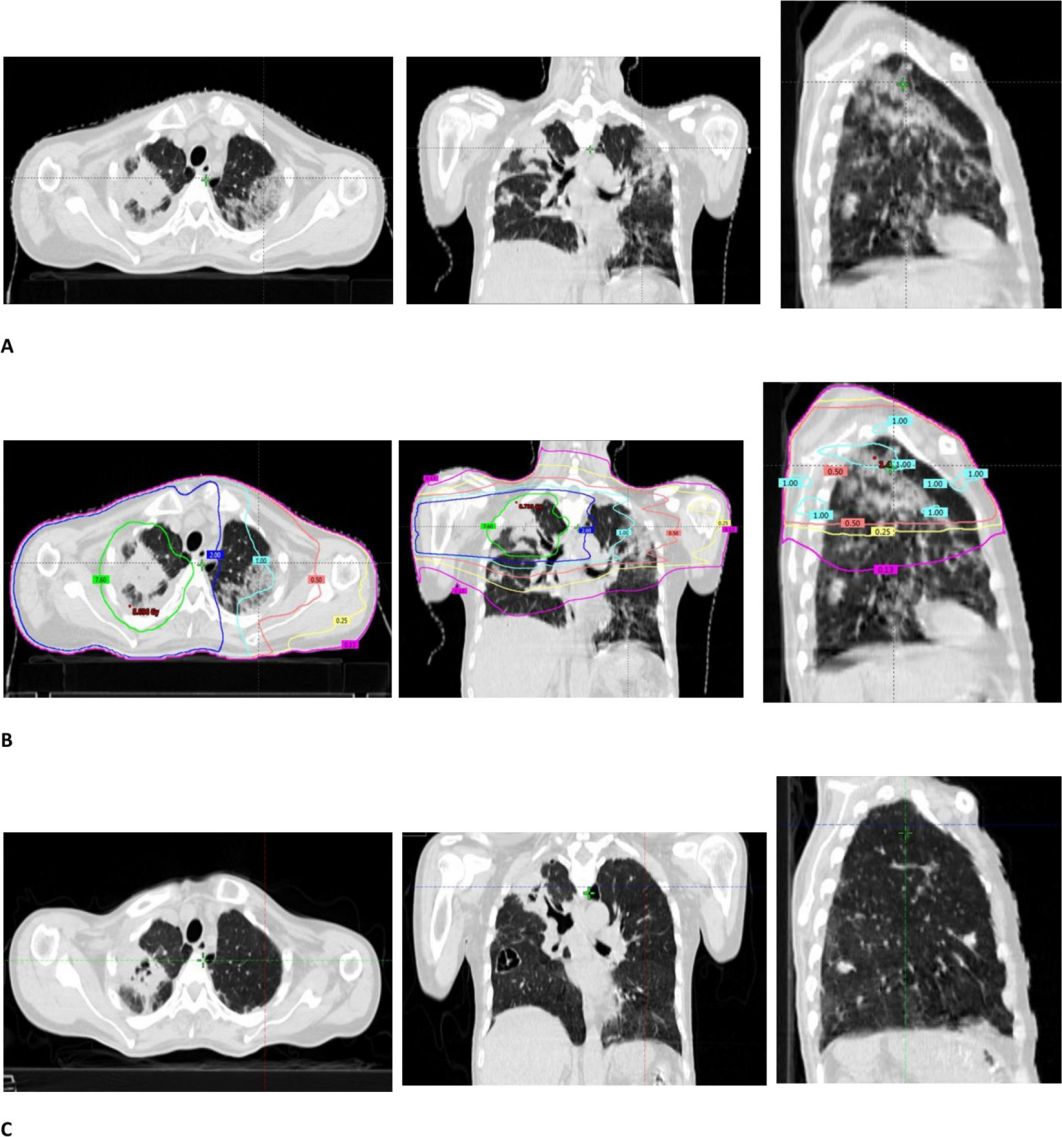
A HRCT scan chest done on 21 November 2020 showed right sided primary lung tumour and bilateral COVID-19 lung lesions (CT-SS 14/25); (**B**) It shows radiation dose distribution around primary lung lesion (Gross tumour volume +5 mm PTV) and low-dose radiation spillage in bilateral lungs. Right-sided upper lobe lesion received palliative radiation – 8 Gy/1 fraction with volumetric modulated arc therapy (VMAT). [Green colour represents (**D_95_**) (7.6 Gy) of 8 Gy delivered to primary lung lesion; Low-dose radiation spillage is represented by dark blue 2 Gy isodose; cyan 1 Gy and pink 0.50 Gy, yellow 0.25 Gy and magenta 0.13 Gy in different areas of both the lungs]; (**C,**) 1-week post-radiation therapy response in bilateral COVID lung lesions attributed to low-dose radiation spillage and CT-SS score declining to 3/25.

Patient gradually improved and fever subsided. Then, he developed recurrent hemoptysis and HRCT chest was done on 21 November 2020 (5 weeks after detection of COVID-19 infection), which reported that in addition to primary lesion, it also showed patchy areas of alveolar opacification/consolidation with surrounding ground glass haze in left upper lobe and lingula. Similar areas were seen in peripheral distribution in right upper, middle and left lower lobe consistent with COVID-19 pneumonia with CT severity score (CT-SS) 14/25 and CORADS (6)^6^ (Figure 1A). A detailed discussion was held amongst oncologists and patient’s family regarding delivering palliative radiation to right lung mass in the presence of COVID-19 lung lesions. After informed consent, a single fraction radiation of 8 Gy was delivered to right upper lobe tumor on 27 November 2020. It was planned with volumetric modulated arc therapy (VMAT) technique to achieve tight dose distribution around the tumor. An attempt was made to minimise dose spillage to surrounding lung by making a shell of 3 cm around PTV with 3 mm margin. However, no extra efforts were made to reduce the low-dose spillage. Tumour dose (D_95_) and low-dose spillage to bilateral lungs (0.13–2 Gy) is depicted in Figure 1B. Mean lung dose was 1 Gy.

## Outcome and follow-up

He had no noticeable side effects with radiation delivered. He had prompt response in haemoptysis and was discharged from the hospital after 3 days. A repeat HRCT done 1 week after radiation showed remarkable resolution of the consolidative changes and ground glass haziness in the previously involved regions of the bilateral lungs with CT severity score declining to 3/25 (Figure 1C). Follow-up CT scans done 3 and 4 weeks later showed mild regression in right lung mass with no change in rest of the findings and was afebrile with no cough, hemoptysis and breathlessness.

He was scheduled to receive systemic chemotherapy or targeted therapy depending upon the status of EGFR, Alk and ROS one mutation studies.

## Discussion

There is paucity of data on patients presenting with lung cancer who also have COVID-19 pneumonia. Also the impact of COVID-19 pneumonia on treatment of lung cancer is not widely available, however, there is a report of higher mortality in lung cancer patients with COVID-19 pneumonia than general population.^3^ Lung cancer patients usually present in locally advanced stages with fast deteriorating symptoms like dyspnoea, cough, hemoptysis necessitating prompt treatment.

COVID-19 patients suffer long-term lung and heart damage but this tends to improve over time according to the data presented at the European Respiratory Society International Congress.^4^ This Austrian study recruited 86 consecutive corona virus patients and they were evaluated at 6, 12 and 24 weeks. At first visit, more than 50% of patients had at least one persistent symptom like breathlessness and cough. CT scans of thorax at 6 weeks showed lung damage in 88% of patients and at 12 weeks, still 56% patients had persistent COVID-19 lung lesions.^4^ Patient in this case study was detected with COVID-19 pneumonia 5 weeks after contracting COVID-19 infection. Long-term respiratory complications of COVID-19 include chronic cough, fibrotic lung disease, bronchiectasis and pulmonary vascular disease.^7^

Viral pneumonia such as COVID-19-induced pneumonia arises since viruses trigger immune cells to synthesize pro-inflammatory cytokines and chemokines inciting the immune response.^8,9^ Recently, low-dose radiation therapy (LDRT) is being examined as therapeutic modality for COVID-19 viral pneumonia as suggested by Kirby and Mackenzie.^5^ Low-dose of radiation (<1 Gy) incites anti-inflammatory properties such as decreasing levels of pro-inflammatory cytokines like IL-1β or inhibiting leukocyte recruitment.^10,11^ It has been argued that low-dose radiation therapy (LDRT) treatment of 30–100cGy (0.3–1.0 Gy) to the lungs of a patient with COVID-19 pneumonia could reduce inflammation and relieve life threatening symptoms.^5^ This dose of radiation to bilateral lungs is very much feasible and would present a very low risk to COVID-19 pneumonia patients.^5^

There are studies that have reported the beneficial effects of LDRT for moderate-to-severe cases of COVID-19 pneumonia during its acute phase (Cytokine storm) (Table 1).^12–14^ However, use of LDRT in patients with persistent COVID pneumonia has not been reported so far.

**Table 1.** Clinical studies of low-dose radiation therapy (LDRT) in COVID-19 pneumonia

S.No.	Study	Institution/Location	No. of patient	Dose and fractions	Inclusion criteria	Response
1	Ameri A et al, 2021^12^	Imam Hossein Hospital Tehran, Iran, Islamic Republic of Iran	10	0.5 Gy radiation to both lungs; may be another fraction of 0.5 Gy (maximum 1 Gy in two fractions at least 72 h apart)	Moderate pulmonary involvement (SPO2 <93% on room air or RR >30/min)	Response rate - 63.6% Clinical recovery - 55.3%
2	Hess CB et al, 2020^13^	Emory University Hospital Midtown/Winship Cancer Institute, Atlanta, GA, USA	5	Single fraction of 1.5 Gy whole lung low-dose radiation therapy	Radiographic consolidation required supplemental oxygen clinically deteriorating	Within 24 h radiographic improvement - 80% Weaned of supplemental oxygen - 60% (one patient improved after 96 h overall recovery - 80%) Mean time to clinical recovery – 35 h
3	Sanmamed N et al, 2021^14^	Clinico San Carlos Hospital, Madrid, spain.	9	Single fraction of 1 Gy	Moderate to severe risk baseline SPO2 - 86–95%	7/9 patients improved 2/9 died

RR – Respiratory rate; SPO2 - Oxygen Saturation

The patient in the present case study, presented with carcinoma of right upper lobe lung with solitary brain metastasis and subsequently developed bilateral COVID-19 pneumonia. Five weeks later, after recovery from COVID-19 symptoms, he developed recurrent hemoptysis and was planned for palliative radiation to right upper lobe lung tumour. The planning CT scan showed typical manifestations of COVID-19 pneumonia involving bilateral lungs. Diagnostic sensitivity of CT scan to accurately diagnose COVID-19 pneumonia is as high as 97% and since these lesions can persist in more than 50% of cases beyond 12 weeks, these new lung lesions were considered to be the manifestations of persisting COVID-19 pneumonia.^4,6^ The patient showed remarkable and quick response in lesions of COVID-19 pneumonia bilaterally conforming to low-dose spillage while delivering therapeutic radiation to right upper lobe tumor with VMAT technique. VMAT technique has many dosimetric advantages. It provides excellent target volume coverage with good organs at risk (OAR) sparing, but it may inadvertently allow a large low-dose bath in surrounding healthy organs/tissues.^15^ COVID-19 lesions in left lower lobe of lung also showed regression with the dose ranging from 0.08 to 0.25 Gy.

In our patient, variable low-dose radiation spillage received by different areas of both the lungs ranged from 0.13 to 2 Gy with VMAT technique (Figure 1B). HRCT chest done after 1 week showed remarkable regression in COVID-19 lesions in the lung bilaterally without any significant morbidity. HRCT chest was repeated at 4 and 5 weeks after radiation, which showed no further significant changes in lung parenchyma. It substantiates the observation made by several authors regarding potential benefit of LDRT on COVID-19 lesions by exerting its anti-inflammatory properties.^9–14^

Dose range (>2 Gy) used in cancer radiotherapy would choose highest achievable dose to obtain maximum tumour response which is pro-inflammatory.^5^ However, LDRT is anti-inflammatory, therefore, while highest dose of radiation would be desirable to the tumor, one has to exploit advanced radiation techniques like intensity-modulated radiation therapy (IMRT) and VMAT to achieve minimum dose to rest of the lung harbouring COVID-19 pneumonia.

In the present case study, while therapeutic radiation of 8 Gy/1 fraction was delivered to right lung tumour for recurrent hemoptysis, low-dose spillage in bilateral lungs yielded significant regression of lesions of COVID-19 pneumonia. The patient received steroids (Dexamethasone 8 mg once daily for 5 days) till 7 November 2020. However, the planning CT scan done on 21 November 2020 showed features of COVID-19 pneumonia bilaterally with CTSS of 14/25. No steroids were given after 7 November 2020 which rules out the possible impact of steroids on COVID-19 pneumonia lesions.

This report gives some degree of evidence regarding favourable response of persisting COVID-19 pneumonia lesions with LDRT. After a thorough literature search including PubMed, Cochrane Library, Medline and Google scholar, we could not find a similar case of favourable response in COVID lesions in a patient of lung cancer treated with therapeutic dose of radiation. This case report may be the first such report in the literature.

We are likely to see many more such patients in near future, since pandemic is still ongoing and cancer of the lung is one of the most frequent cancers worldwide.^1,2^ This case study would help in offering therapeutic radiation to many more patients of lung cancer who develop persisting manifestations of COVID-19 pneumonia. However, it would be desirable to have experience of larger number of patients and preferably a prospective study to understand the timing of radiation therapy and dose–response relationship of LDRT to achieve maximum benefit.

## Conclusion

Low-dose scatter radiation in bilateral lungs resulted in near total resolution of COVID-19 pneumonia lesions, while delivering palliative radiation to right upper lobe lung carcinoma with VMAT technique.

## Learning points

1. COVID-19 pneumonia lesions can persist for more than 3–6 months after COVID-19 infection.
2. It is possible to deliver therapeutic radiation to lung tumor in the presence of persisting COVID-19 pneumonia provided the mean dose to rest of the lung is kept below 1 Gy.
3. Low-dose radiation therapy yielded near total resolution of persisting COVID-19 pneumonia lesions.
